# The Effect of Atopic Dermatitis and Diet on the Skin Transcriptome in Staffordshire Bull Terriers

**DOI:** 10.3389/fvets.2020.552251

**Published:** 2020-10-16

**Authors:** Johanna Anturaniemi, Sara Zaldívar-López, Huub F. J. Savelkoul, Kari Elo, Anna Hielm-Björkman

**Affiliations:** ^1^Faculty of Veterinary Medicine, Department of Equine and Small Animal Medicine, University of Helsinki, Helsinki, Finland; ^2^Genomics and Animal Breeding Group, Department of Genetics, Faculty of Veterinary Medicine, University of Córdoba, Córdoba, Spain; ^3^Cell Biology and Immunology Group, Wageningen University, Wageningen, Netherlands; ^4^Faculty of Agriculture and Forestry, Department of Agricultural Sciences, University of Helsinki, Helsinki, Finland

**Keywords:** atopic dermatitis, canine, diet, gene expression, RNAseq, skin, kibble diet, raw meat-based diet

## Abstract

Canine atopic dermatitis (CAD) has a hereditary basis that is modified by interactions with the environment, including diet. Differentially expressed genes in non-lesional skin, determined by RNA sequencing before and after a dietary intervention, were compared between dogs with naturally occurring CAD (*n* = 4) and healthy dogs (*n* = 4). The dogs were fed either a common commercial heat-processed high carbohydrate food (kibble diet) (*n* = 4), or a non-processed high fat food (raw meat-based diet) (*n* = 4). At the end of the diet intervention, 149 differentially expressed transcripts were found between the atopic and healthy dogs. The main canonical pathways altered by the dysregulation of these genes were angiopoietin signaling, epidermal growth factor signaling, activation of angiogenesis, and alterations in keratinocyte proliferation and lipid metabolism. On the other hand, 33 differently expressed transcripts were found between the two diet groups, of which 8 encode genes that are annotated in the current version of the dog genome: immunoglobulin heavy constant mu (*IGHM*), immunoglobulin lambda-like polypeptide 5 (*IGLL5*), B-cell antigen receptor complex-associated protein beta chain (*CD79B*), polymeric immunoglobulin receptor (*PIGR*), cystathionine β-synthase (*CBS*), argininosuccinate synthase 1 (*ASS1*), secretory leukocyte peptidase inhibitor (*SLPI*), and mitochondrial ribosome recycling factor (*MRRF*). All genes were upregulated in the raw diet group. In conclusion the findings of this study suggest alterations in lipid and keratinocyte metabolism as well as angiogenesis in the skin of atopic dogs. Additionally, a possible enhancement of innate immunity and decrease in oxidative stress was seen in raw food fed dogs, which could have an important role in preventing hypersensitivities and disturbed immunity at young age.

## Introduction

Atopic dermatitis (AD) in humans is a complex hereditary disease characterized by its genetic pre- disposition, as well as how it causes immunologic hyperreactivity and a defective skin barrier. An impaired epidermal barrier is one of the typical features of AD (1–3). Also, environmental factors play a role in its pathogenesis (4). Canine atopic dermatitis (CAD) shares genetic, phenotypic, and histological similarities with human AD (5). CAD is estimated to affect 10–15% of the canine population and seems to be increasing over time (5, 6). It has been reported that pure bred dogs are more likely to develop atopy and allergic dermatitis than mixed breeds (7). Staffordshire bull terriers and similar breeds (bull terrier, American Staffordshire terrier, and pit bull terrier) have previously been shown to be particularly susceptible to CAD (8–13).

Diet has profound effects on the whole body, including its metabolism. People with skin conditions often modify their diet to influence their clinical symptoms (14–16), as do dog owners for their dogs (17). It has been previously suggested that clinical manifestations of CAD can be modulated by diet (18–21). It has been reported that certain diets can potentially affect skin symptoms positively both in humans (22–24) and in dogs (25–27). Research on how diets impact skin gene expression in dogs is still scarce. However, dietary changes are shown to change the gene expression in the skin of mice (28), rats (29), and humans (30, 31). The impact that the elimination diet has on the skin of dogs with cutaneous adverse food reactions has been studied using quantitative PCR (qPCR) (32). The diets influence on gene expression in the liver (33), skeletal muscle (34), adipose tissue (35), colonic mucosa (36), and brain tissue (37) of dogs has also been studied.

Previously published studies that have focused on altered gene expression in atopic canine skin have used mRNA microarrays (38–40) and (qPCR) (41–50). The objective of this study was to find differentially expressed genes between atopic and healthy dogs, as well as to compare the effects of two different diet types on the gene expression of the skin using RNA sequencing (RNA-Seq). As alterations in skin function of atopic patients are already well recognized, our hypothesis was that there would be differences in the gene expression between atopic and healthy dogs. In addition, we hypothesized that two different dietary choices would subsequently affect skin gene expression differently. Eight Staffordshire bull terriers were used, both atopic and healthy, equally distributed between diet groups. To our best knowledge, neither previous studies using RNA-Seq to compare atopic and healthy dogs, or studies regarding the effect of diet on canine skin gene expression have been conducted.

## Methods

### Animals and Sample Collection

The study protocol was approved by the Animal Experiment Board in Finland (ELLA) (permit number: ESAVI/3244/04.10.07/2013). All owners filled in and signed a written consent form. Dogs were living in their home environment during the diet intervention trial. The eight study dogs used in the present study were part of a larger diet intervention trial studying CAD. The study was conducted at the department of equine and small animal medicine at the University of Helsinki.

At the baseline visit, dogs were evaluated by a veterinarian and blood samples were collected. Non-lesional skin biopsies were taken from the axillary area under anesthesia using an 8 mm biopsy punch, and samples were immediately stored at −80°C. The animals were sedated using dexmedetomidine (Dexdomitor, Orion Pharma) 5–10 μg/kg intramuscularly and intravenous propofol (PropoVet, Orion Pharma) as needed. Skin biopsies were taken only from dogs that had not taken oral glucocorticoids and cyclosporine for 4 weeks, or oral antihistamines, topical glucocorticoids, and medicated shampoos for 2 weeks prior to sample collection.

After the baseline visit, the dogs were divided into two diet groups, and fed either a commercial heat-processed, high carbohydrate (kibble) diet [Hill's Science PlanTM Canine Adult Sensitive Skin with Chicken (KD), detailed composition of food shown in Supplementary Table 1], or a commercial non-processed, high fat (raw meat-based) diet [MUSH BARF Vaisto® Pork-Chicken-Lamb and/or MUSH BARF Vaisto® Beef-Turkey-Salmon (RMBD), detailed compositions shown in Supplementary Table 2]. The diets differed by their fat and carbohydrate content, their ingredients (KD having chicken as a main animal protein source and RMBDs having three different animal protein sources each) and their manufacturing methods (the KD was heat-processed and the RMBDs were only ground and frozen). Owners were asked to feed their dogs at least 99.9% trial diet, giving portions as recommended by the manufacturer, adjusting if necessary, to maintain normal body weight. Water was allowed *ad libitum*. At the end visit, the same protocol was followed as the baseline visit, and non-lesional skin biopsies were again obtained from the same area and immediately stored at −80°C.

Skin biopsies were obtained from client-owned Staffordshire bull terriers (*n* = 8). They were stratified between diet cohorts by age, sex, and health status when possible, since the number of healthy dogs was very small. The dogs diagnosed with naturally occurring CAD (*n* = 4) were fed either the KD (*n* = 2) or RMBD (*n* = 2). The healthy control dogs were fed either the KD (*n* = 2) or the RMBD (*n* = 2). This allowed comparisons to be made between KD-fed (*n* = 4) and RMBD-fed (*n* = 4) dogs, as well as between atopic (*n* = 4) and healthy (*n* = 4) dogs. Information regarding the dogs chosen for RNA-Seq analyses (*n* = 8) are shown in Table 1.

**Table 1:** Basic information of the eight dogs used in RNA-Sequencing analyses.

**Dog ID**	**Age (years)**	**Gender**	**Diet group**	**Diagnosis**
55	13	Female	KD	Healthy
47	4	Male	KD	Healthy
66	4	Male	KD	CAD
33	4	Female	KD	CAD
65	3	Female	RMBD	Healthy
37	5	Female	RMBD	Healthy
40	6	Female	RMBD	CAD
34	2	Male	RMBD	CAD

*KD, kibble diet; RMBD, raw meat-based diet; CAD, canine atopic dermatitis*.

### RNA Extraction

Prior to RNA extraction, the skin biopsy samples were transferred to RNAlater®-ICE Frozen Tissue Transition Solution (Life Technologies, Carlsbad, CA, USA) and allowed to thaw overnight at −20°C. Subcutaneous fat was carefully trimmed from the skin biopsies, and the samples were homogenized using a tissue homogenizer (TissueRuptor, Qiagen, Hilden, Germany). Total RNA was extracted using Qiagen miRNAeasy Mini Kit (Qiagen, Hilden, Germany) according to the manufacturer's recommended protocol. After RNA extraction, DNAse treatment using RNase-free DNase I (Thermo Fisher Scientific, Waltham, MA, USA) was performed. The total RNA concentration of samples was analyzed using a 260 nm ultraviolet spectrophotometer (NanoDrop ND-1000, Thermo Fisher Scientific, Wilmington, DE, USA). The integrity and quality of the RNA was analyzed using an Agilent 2100 Bioanalyzer (Agilent Biotechnologies Inc., Santa Clara, CA, USA) and only samples with RIN >7 and RNA amount higher than 1 μg were sent for analysis. Prior to being sent to the sequencing facility, the samples were stored at −80°C.

### RNA-Seq and Data Analysis

Next generation sequencing was performed on all of RNA skin samples of the dogs, taken at the baseline (*n* = 8) and end of the diet intervention (*n* = 8). One microgram (μg) of total RNA was ribodepleted and an RNA-Seq library was created using a ScriptSeq v2™ Complete kit for human/mouse/rat (Illumina, Inc., San Diego, CA, USA). Paired-end library creation and transcriptome sequencing were completed at the Institute for Molecular Medicine Finland (FIMM). Libraries were quality controlled by High Sensitivity chips by Agilent Bioanalyzer (Agilent) before being sequenced on an Illumina HiSeq platform (HiSeq 2000, Illumina, Inc., San Diego, CA, USA).

The bioinformatic analysis was performed at the Plataforma Andaluza de Bioinformática (University of Málaga, Spain). Quality control and initial pre-processing was performed using SeqTrimNext (v. 2.0.53), where low quality, ambiguous, low complexity stretches, adaptor, organelle DNA, polyA/polyT tails, and contaminating sequences were removed (51). Mapping was performed with restrictive conditions of bowtie2 (v.2.2.2), including parameters for rejecting discordant alignments and optimized for paired-end reads (52). Samtools (v.0.1.19) quantified known transcripts (count reads per transcripts) (53), and transcripts were annotated using Full-Lengther-Next (54). Statistical comparisons of transcript expression between diet groups and between atopic and healthy dogs were performed using DEgenes-Hunter (v.2.0.11) (55), a tool that imputes raw read counts generated by Bowtie2/Samtools into the EdgeR (56) and DESeq2 (57) algorithms. Fold change (FC) ≥2, and a false discovery rate (FDR) corrected *p* < 0.05 were set as thresholds. Differentially expressed transcripts common to both analyses (EdgeR and DESeq) were also identified. RNA-Seq can detect a higher percentage of differentially expressed genes compared to expression arrays, especially genes with low abundance. Typically, in many controlled experiments which utilize RNA-Seq, the number of biological samples is a limiting factor (57) which ultimately restricts the statistical inference to the largest gene expression differences between the groups. Specific algorithms have been developed to improve statistical inference in RNA-Seq datasets with small sample sizes (56–58). EdgeR and DESeq2 are shown to have the highest sensitivity to detect true differences between group means in read count datasets produced by RNA-Seq methods (57, 58). These tools utilize different approaches in calculation of dispersion estimates for read count datasets, and algorithms are differently affected by outlying observations (58).

The comparisons related to dogs' characteristics between the two diet groups were analyzed using the Mann-Whitney U test, Fisher's Exact test and the Wilcoxon signed ranks test. SPSS software (version 25, IBM SPSS Statistics. Chicago, Ill., USA) was used for the statistical analyses. The statistical significance threshold was set at *P* < 0.05.

## Results

### Clinical Findings

There were no statistical differences between the comparison groups (*n* = 8) in any of the recorded descriptive data of the dogs (Tables 2, 3). The mean weight of the dogs in the atopic (*n* = 4) and healthy cohorts (*n* = 4) did not change significantly during the trial (*p* = 0.180 and 0.141, respectively; Table 2), or in the KD-fed and RMBD-fed cohorts (*p* = 0.285 and 0.102, respectively; Table 3). The diet intervention lasted 84–147 days (median 137 days).

**Table 2:** Descriptive data and statistical analysis of the atopic and healthy dogs used in the study comparing the skin gene expression profiles (*n* = 8).

		**Atopic dogs**	**Healthy dogs**	***p*-value**
Dogs	*N*	4	4	
Age	Years	3.5 (±1.7)	6.0 (±4.7)	0.468
Gender	% male	50	25	0.500
Duration	Days, median	129	146	0.058
Weight	Baseline, Kg	17.3 (±2.9)	16.1 (±1.8)	0.773
	End visit, Kg	17.7 (±2.7)	16.7 (±1.7)	0.564

*Data are mean ± SD. Analyzed using Mann-Whitney U-test, Fisher's Exact test and Wilcoxon signed ranks test*.

**Table 3:** Descriptive data and statistical analysis of the kibble diet (KD) and raw meat-based diet (RMBD) fed dogs used in the study comparing the skin gene expression profiles (*n* = 8).

		**KD group**	**RMBD group**	***p*-value**
Dogs	*N*	4	4	
Age	Years	5.9 (±4.8)	3.6 (±1.8)	0.663
Gender	% male	50	25	0.500
Duration	Days, median	144	132	0.465
Weight	Baseline, Kg	16.3 (±1.4)	17.1 (±3.3)	0.773
	End visit, Kg	16.7 (±1.2)	17.7 (±2.9)	0.564

*Data are mean ± SD. Analyzed using Mann-Whitney U-test, Fisher's Exact test and Wilcoxon signed ranks test*.

### Sequencing Overview

An average of 52.2 million sequencing reads were obtained per sample, ranging from 40.7 to 69.4 million reads. Mean read length obtained from sequencing was 93.8 bp after pre-processing quality trimming. An average of 93.47% of the reads were mapped to the canine reference genome. The obtained read counts were then uploaded into the DEgenes-Hunter tool, where comparisons using two different software (DESeq2 and EdgeR) were performed.

### Differential Gene Expression Between Atopic and Healthy Dogs

At the baseline visit, three downregulated genes were found in atopic dogs compared to healthy dogs by EdgeR. Four downregulated genes and one upregulated gene were found by DESeq2 in atopic dogs compared to healthy dogs (Table 4).

**Table 4:** Differentially expressed genes in the skin of atopic dogs (*n* = 4) compared to healthy dogs (*n* = 4) at the baseline visit.

**Gene**	**FDR**	***P*-value**	**Log_**2**_FC**	**Algorithm**
PKHD1	0.0242	6.00E-06	−2.6	EdgeR
KRT4	0.0013	1.57E-07	−4.8	EdgeR
LYZF2^*^	0.0032	5.92E-07	−4.6	EdgeR
AHDC1	0.0168	5.35E-06	−0.73	DESeq2
DEDD	0.0259	9.89E-06	−0.60	DESeq2
SGOL2	0.0146	2.05E-06	0.66	DESeq2
LEPR*	0.0295	4.60E-05	−1.1	DESeq2
DUSP1	0.0297	1.31E-05	−0.87	DESeq2

RNA-sequensing was used.

* *Multiple transcripts were found, only the one with the highest FC are shown. FDR, false discovery rate corrected p-value; PKHD1, polycystic kidney and hepatic disease 1; KRT4, keratin 4; LYZF2, lysozyme C; AHDC1, AT-hook DNA-binding motif-containing protein 1; DEDD, death effector domain containing, SGOL2, shugoshin 2; LEPR, leptin receptor; DUSP1, dual specificity phosphatase 1*.

**Figure 1:**
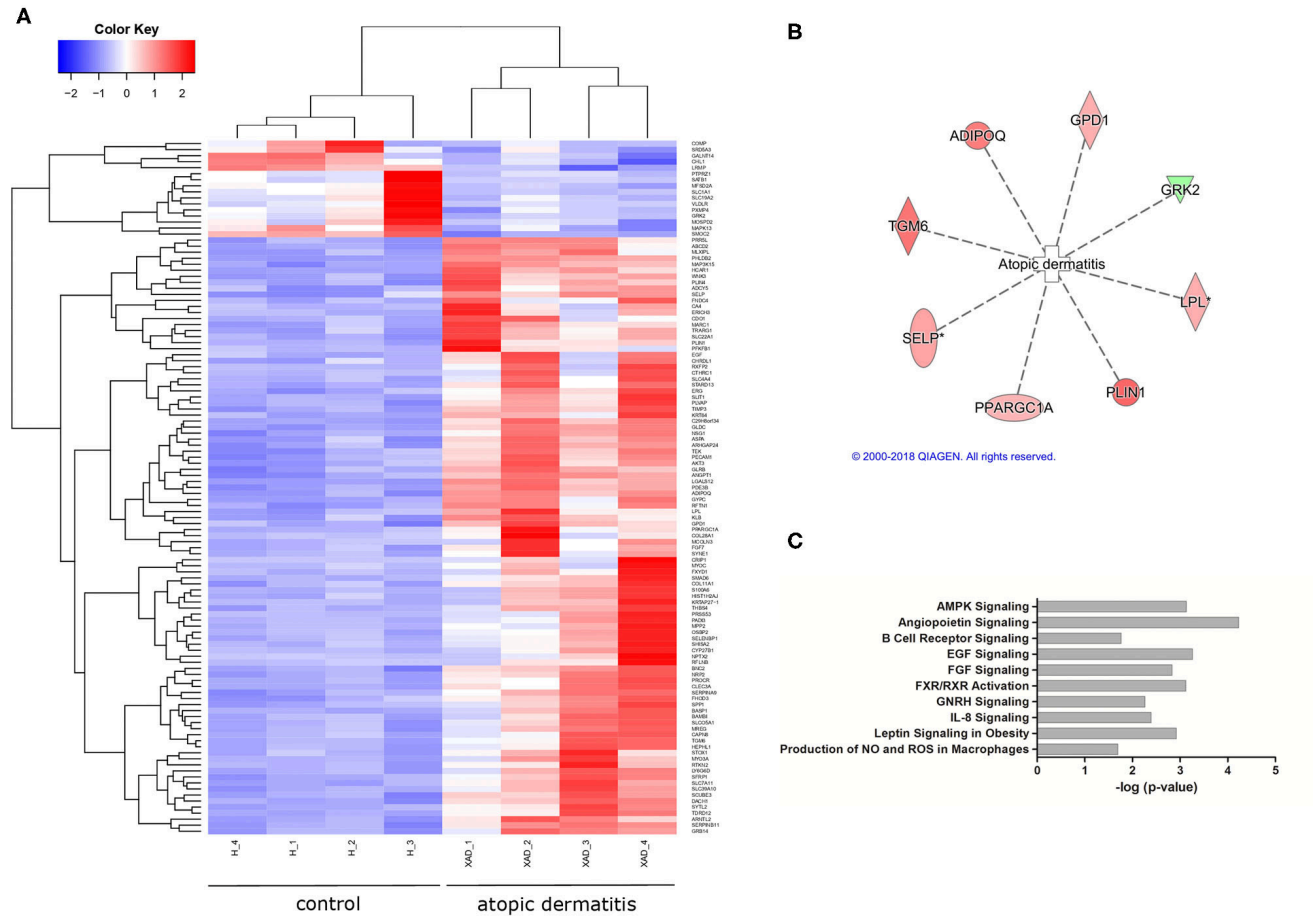
Differential gene expression in atopic vs. healthy dogs (*n* = 8). **(A)** A heatmap showing differentially expressed genes in atopic and healthy dogs (downregulated in blue, upregulated in red). **(B)** A total of eight genes associated with atopic dermatitis were found differentially expressed in atopic dogs after the diet trial (green indicates downregulation, red indicates increased expression). **(C)** Signaling pathways affected by gene dysregulation in dogs with atopic dermatitis compared to healthy dogs.

After the diet intervention, EdgeR found 200 transcripts, and DeSeq found 451 transcripts that were differentially expressed between atopic and healthy groups after the diet intervention (S3), of which 149 differentially expressed transcripts between groups were found by both EdgeR and DESeq2 (Figure 1A; Supplementary Table 3). According to the analyses of their biological function, 69 of the differentially expressed transcripts were involved in dermatological and inflammatory conditions, of which 8 were associated with atopic dermatitis based on the analysis of biological functions (Figure 1B). Signaling pathways affected by gene dysregulation in the atopic group included angiopoietin signaling, epidermal growth factor signaling, AMP-activated protein kinase signaling, retinoid X receptor (RXR)/farnesoid X receptor (FXR) signaling, and leptin signaling in obesity. Also, upregulation of EGF, AKT3, KLB, ANGPT1, and TEK led to the predicted activation (z-score = 2.23) of the IL-8 inflammatory pathway (Figure 1C).

### Comparison Between the Two Diet Groups

**Figure 2:**
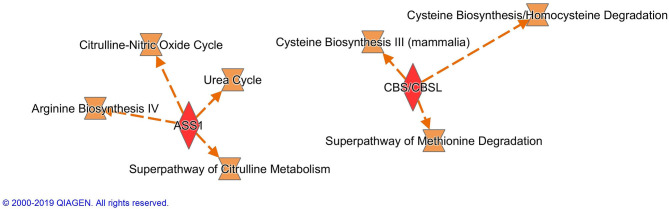
Canonical pathways activated by the expression of ASS1 and CBS in the raw meat-based diet fed dogs. ASS1, argininosuccinate synthase 1; CBS, cystathionine β-synthase.

**Figure 3:**
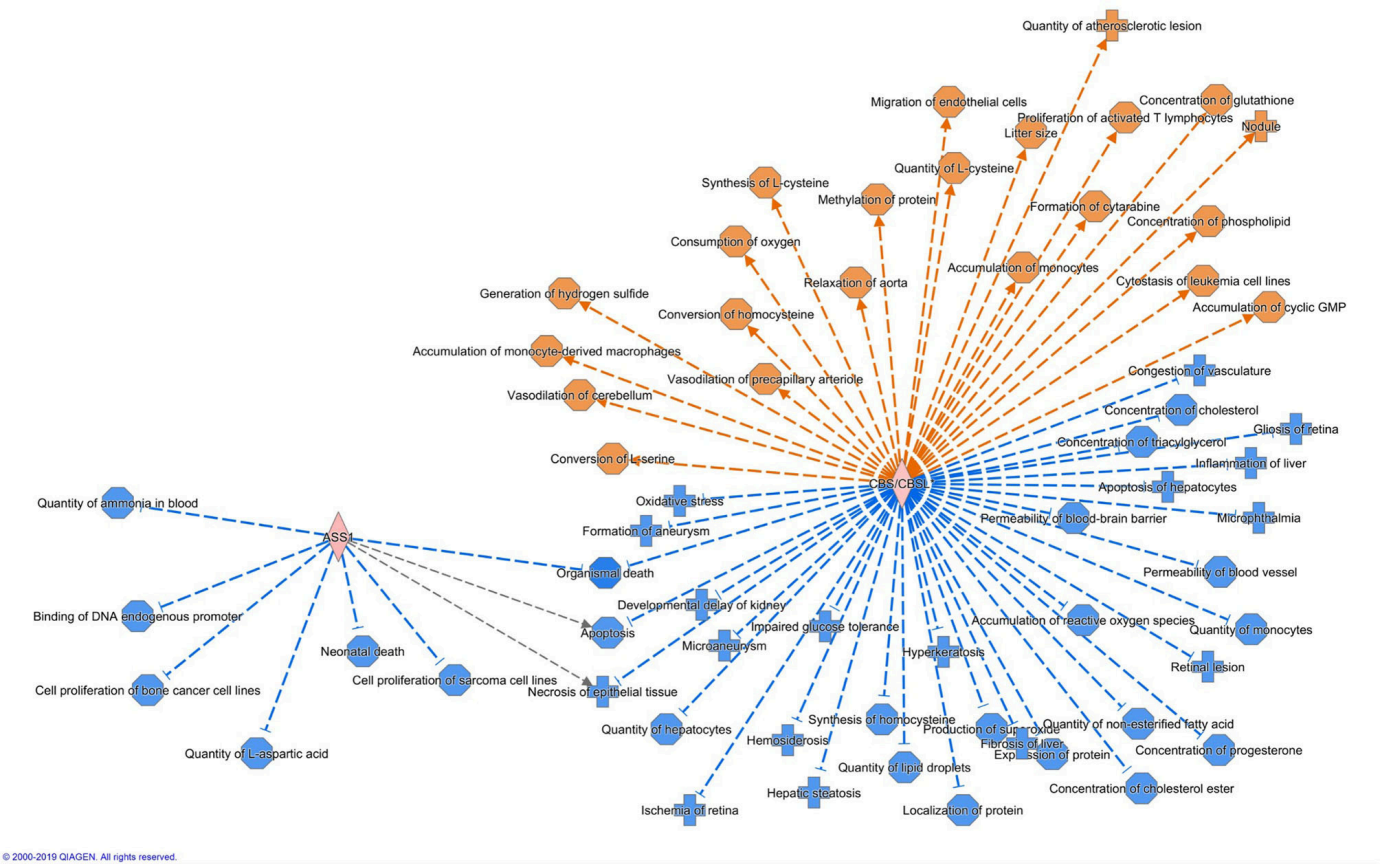
Biological functions activated (in orange) or inhibited (in blue) by the expression of ASS1 and CBS in the raw meat-based diet fed dogs. ASS1, argininosuccinate synthase 1; CBS, cystathionine β-synthase.

**Figure 4:**
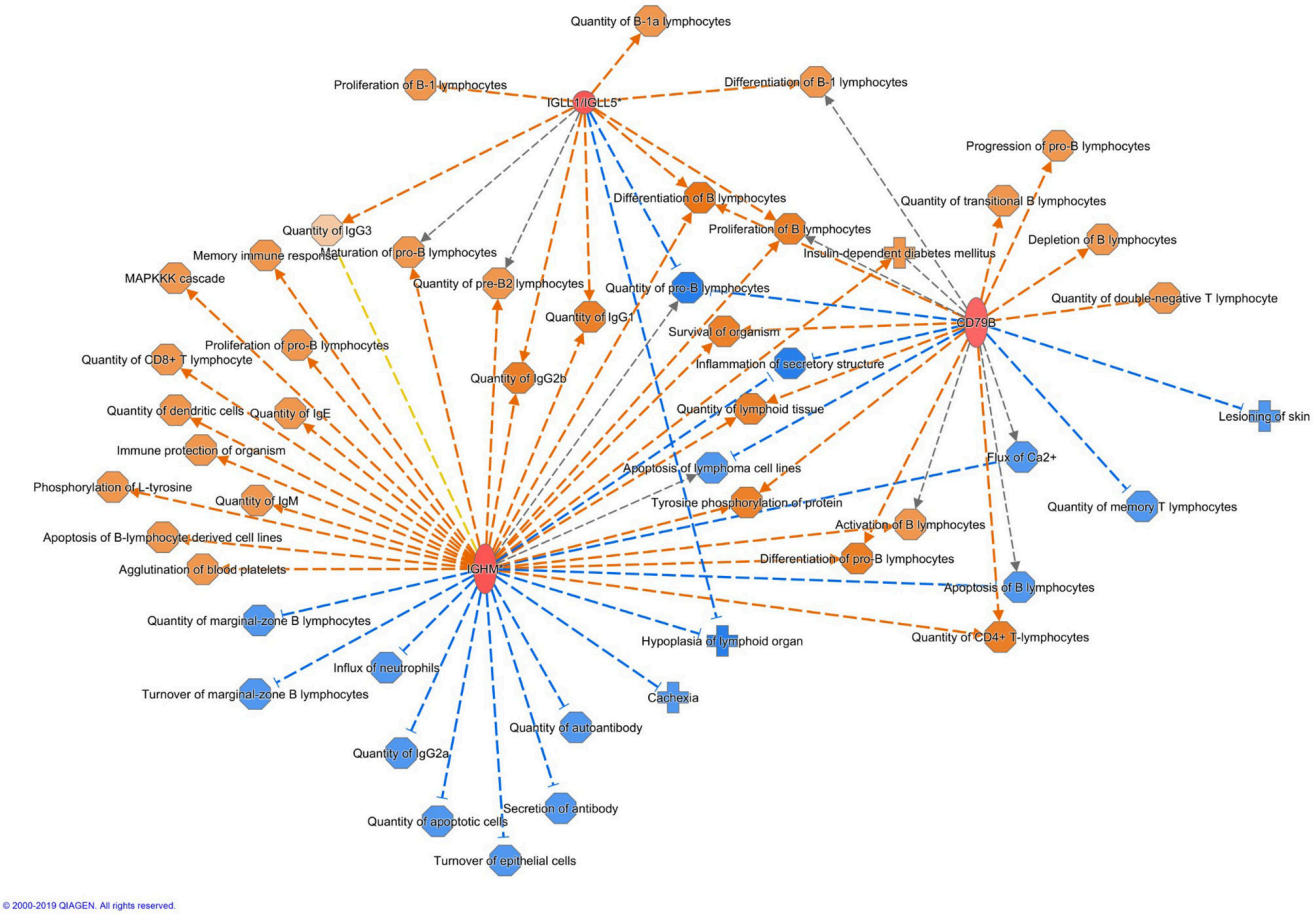
Biological functions activated (in orange) or inhibited (in blue) by the expression of IGHM, IGLL5, and CD79B in the raw meat-based diet fed dogs. IGHM, immunoglobulin heavy constant mu; IGLL5, immunoglobulin lambda-like polypeptide 5; CD79B, B-cell antigen receptor complex-associated protein beta chain.

Altogether 33 differently expressed transcripts were found between the diet groups, of which 8 genes are annotated in the current version of the dog genome: immunoglobulin heavy constant mu (*IGHM*), immunoglobulin lambda-like polypeptide 5 (*IGLL5*), B-cell antigen receptor complex-associated protein beta chain (*CD79B*), polymeric immunoglobulin receptor (*PIGR*), cystathionine β-synthase (*CBS*), arginosuccinate synthase 1 (*ASS1*), secretory leukocyte peptidase inhibitor (*SLPI*), and mitochondrial ribosome recycling factor (*MRRF*). These genes (*n* = 8) were all upregulated in the RMBD group compared to the KD group (Table 5). They were then studied regarding their biological implication. Activation of ASS1 and CBS in the raw diet group indicated upregulation of cystein biosynthesis and methionine degradation, the citrulline-nitric oxide cycle, the urea cycle, and arginine biosynthesis (Figure 2). Biological functions activated by CBS included for example concentration of glutathione, conversion of homocysteine, and concentration of phospholipids, and inhibited by CBS included for example oxidative stress, hyperkeratosis, and accumulation of reactive oxygen species (Figure 3). In addition, the expression of IGHM, IGLL5, and CD79B activates the differentiation of B lymphocytes and the memory immune response, determines the quantity of different immunoglobulins, inhibits hypoplasia of the lymphoid organ, determines the quantity and secretion of autoantibody, inflammation of secretory structure, and lesioning of skin (Figure 4).

**Table 5:** Differentially expressed genes in raw meat-based diet fed dogs compared to kibble diet fed dogs after the diet intervention (*n* = 8).

**Gene name**	**FDR**	**P-value**	**Log_**2**_FC**	**Algorithm**
PIGR	0.0225	2.48E-05	6.4	EdgeR
SLPI	0.0487	6.11E-05	5.4	EdgeR
IGHM^*^	1.28E-05	7.83E-10	5.3	ER/ DS
IGLL5^*^	0.00287	1,56E-06	5.3	
CD79B	0.00564	5.09E-06	4.8	
ASS1	0.00564	5.17E-06	2.1	ER/ DS
CBS^*^	0.0408	4.74E-05	1.8	ER/ DS
MRRF	0.0360	2.26E-05	0.70	DESeq2

Only genes that are annotated in the dog genome are shown.

* *Multiple transcripts were found, only the one with the highest FC are shown. FDR, false discovery rate; FC, fold change; PIGR, polymeric immunoglobulin receptor; SLPI, secretory leukocyte peptidase inhibitor; IGHM, immunoglobulin heavy constant mu; ASS1, argininosuccinic synthase 1; CBS, cystathionine β-synthase; MRRF, mitochondrial ribosome recycling factor; IGLL5, immunoglobulin lambda-like polypeptide 5; CD79B, B-cell antigen receptor complex-associated protein beta chain; ER, EdgeR; DS, DESeq2*.

The activation of immunological pathways such as IL-7 signaling, and B-cell related pathways were also found. Upregulation of (natural) IgM antibodies can result from enhanced IL-7 production by epithelial cells in the gastro-intestinal tract, where they are then shuttled across the epithelium into the lumen as a result of upregulated poly Ig receptor expression (59).

## Discussion

To our best knowledge, the present study is the first RNA-Seq study of altered skin gene expression between dogs fed two different diets. Although the sample sizes in this study was limited, differences were found both between the atopic and healthy groups and between the diet groups. There were many more differentially expressed genes found between the same atopic and healthy dogs at the end of the diet intervention than at baseline. This suggests that the diet is an important background factor which should be considered when studying the gene expression of animals with skin diseases.

In dogs suffering from CAD, the defective skin barrier is believed to facilitate the penetration of allergens into the skin. This then leads to sensitization against environmental allergens and subsequent cutaneous inflammation, which then further aggravates the impairment of the skin barrier (60). Lipids in the skin are important to its barrier function as they help prevent transepidermal water loss (TEWL) (61). Expression of galectin 12 (*LGALS12*), hydroxycarboxylic acid receptor 1 (*HCAR1*) and ATP Binding Cassette Subfamily D Member 2 (*ABCD2*) were upregulated in the atopic dogs in the present study. *LGALS12* and *HCAR1* suppress lipolysis (62, 63). As part of the barrier formation process, keratinocytes secrete lipids through lipolysis (64). Very-long chain fatty acids (VLCFA) act as a water barrier and are important in forming a cornified envelope, both of which help maintain the integrity and functionality of the skin (65, 66). ABCD2 is suggested to have a role in a transporting VLCFAs to peroxisomes for degradation by β-oxidation (67). These results suggest that the lipid metabolism in the skin of atopic dogs might have been impaired, detrimentally affecting skin barrier function.

The cornified envelope formation of the skin also requires the differentiation of keratinocytes (61, 68). This differentiation is regulated by the epidermal differentiation complex (EDC). Another gene cluster located in the EDC is the S100 gene family. A higher expression of S100 calcium binding protein A6 (*S100A6*) was found in the non-lesional skin of atopic dogs compared to healthy dogs. One member of the S100 gene family, *S100A8*, has been previously shown to be associated with lesional skin of atopic dogs (38–50). A recent *in vitro* study showed that the overexpression of *S100A6* results in a less differentiated keratinocyte phenotype and thus disturbs the differentiation process (69). Additionally, one of the epigenetic regulators needed for the terminal differentiation of keratinocytes, special AT-rich sequence-binding protein 1 (*SATB1*) (70), was downregulated in the skin of atopic dogs in the present study. These results indicate that the differentiation of keratinocytes in the skin of atopic dogs might be dysfunctional. Altered gene expression of keratins (KRTs) are found in both the skin of atopic dogs (39, 40, 71) and human AD patients (39, 72). In the present study, a transcript of the *KRT4* gene was highly downregulated, and the *KRT84* gene was overexpressed in all atopic dogs compared to healthy dogs at the diet intervention baseline. An association of *KRT84* with CAD has not been previously reported.

In the present study, angiopoietin signaling was the most upregulated canonical pathway found in the atopic dogs. Angiogenesis has been reported to play a role in atopic dermatitis (73–75). In the present study AKT serine/threonine kinase 3 (*AKT3*), angiopoietin 1 (*ANGPT1*), tyrosine receptor kinase (*TEK*), and secreted phosphoprotein 1 (*SPP1*) were upregulated in the skin of atopic dogs, all of which are known to regulate angiogenesis (76–78). *ANGPT1* has been shown to be upregulated in the skin of the AD mouse model (75). Together with *TEK, ANGPT1* has also been reported to be upregulated in psoriatic skin in humans (79). Elevated expression of *SPP1* in the skin of psoriatic patients has been reported, but the expression of *SPP1* in lesional skin of AD patients was not observed (80). Hence the upregulated angiogenesis found in the present study comports with previous research.

The upregulation of several immunity-related genes was found in the RMBD fed dogs at the end of the diet intervention. The *IGHM* gene encodes the C region of the mu heavy chain, which defines the IgM isotype. As immunoglobulin M's (IgM's) are the first antibodies to be produced in an ongoing immune response to infection or immunization (81), the upregulation of *IGHM* in the RMBD fed group may indicate activation of humoral immune mechanisms. IgM antibodies are generally polyspecific and have low binding affinities and reflect an increased innate immune defense. Together with *IGLL5, IGHM* regulates both the quantity of different immunoglobulins and inhibits the hypoplasia of lymphoid organs. Together with *CD79B, IGHM* increases lymphoid tissue quantity. *CD79B* inhibits biological functions such as skin lesioning and inflammation of secretory structures. Activation of these three genes also increases the proliferation of B lymphocytes. Playing an important role in the mucosal immune system, *PIGR* transports polymeric immunoglobulins to the apical surfaces of epithelia (82). While IgM and IgA antibodies only have a limited antigen specificity, they generally show a large bystander response. Thus, secretory IgMs and IgAs in the gastrointestinal tract are polyreactive against primarily commensal bacteria and most of these “natural” anti-commensal secretory Igs (sIgs) are made through T cell-independent B cell responses (83). Secretions collected at mucosal surfaces contain significant proportions of IgA due to passive transudation, reflecting the degree of mucosal inflammation. The sIgA is generated at the cleavage site of PIGR and acts as an inhibitory factor against bacteria on the skin surface. In human AD, abnormalities in sIgA have been reported (84). Previous studies have indicated that *PIGR* and its secretory component have an anti-inflammatory role in inflammatory skin diseases (85–87).

In the citrulline-nitric oxide (NO) cycle, found in many cells, ASS1 is a rate-limiting enzyme for nitric oxide synthesis (88). The expression of *ASS1* in macrophages and neutrophils can be upregulated in response to bacterial lipopolysaccharides, and hence also contributes to the innate immune defense (89). Although bacteria present in the RMBD may have enhanced the expression of *ASS1*, its expression is also necessary for optimal control of persistent pathogens (90), which may be beneficial to dogs suffering from CAD. NO functions as an antimicrobial (91) and can initiate human keratinocyte differentiation (92), which is necessary for the proper development of a functional skin barrier (93). It has been suggested that high concentrations of NO are functionally important for the resolution of chronic inflammatory processes (42, 94).

*SLPI*, which is a serine protease inhibitor and an antimicrobial peptide, was upregulated in the RMBD group. Lancto et al. (42) found a lower expression of *SLPI* both in the lesional and non-lesional skin of atopic dogs compared to healthy dogs. Considering the roles of *IGHM, PIGR, ASS1*, and *SLPI*, the RMBD may have both enhanced the dogs' innate immunity and improved barrier function of the skin. Consumption of certain dietary constituents found in the RMBD, namely water-soluble vitamins and amino acids, have been previously shown to positively affect the skin barrier by decreasing TEWL in dogs (93). It remains unclear whether the effect seen in the present study was due to the quantity of certain nutrients, or due to the differences in fat or moisture content of the diets.

*CBS* catalyzes a reaction in which serine and L-homocysteine (Hcy) are condensed to cystathionine and subsequently converted to cysteine, which is the limiting reagent in the production of glutathione, an important antioxidant (95–97). Inflammatory processes cause alterations to this pathway (97) and reduced glutathione production is associated with an increased vulnerability to oxidative stress (96). Elevated oxidative stress and immune dysfunction, which eventually leads to skin damage, appears to also play a role in the pathophysiology of atopic dermatitis. Clinical symptoms of atopic dermatitis can thus be mitigated by increasing antioxidant levels, as they help reduce oxidative damage. A study recently reported that cystathionine and cysteine inhibit the upregulation of proinflammatory mediators in human keratinocytes (98). Additionally, *MRRF* depletion results in elevated ROS production and cellular dysfunction (99). In our study, the expression of *CBS* and *MRRF* suggests that the RMBD may have inhibited ROS production in skin cells. Oxidative stress plays an important role in the pathogenesis of atopic dermatitis in mammals (100, 101), and the induction of oxidative stress is related to both excessive levels of ROS and to deficiencies within the antioxidant system (102). The possible effects of increased *CBS* and *MRRF* activity might offer therapeutic value to atopic dogs, since ROS production is increased in canine atopic dermatitis during the inflammatory process (103).

Anderson et al. (104) studied gene expression profiles of peripheral blood mononuclear cells from dogs fed either a kibble diet (*n* = 8) or a raw red meat diet (*n* = 7) using Agilent Canine 4 × 44 k microarrays. Their results indicated that a short-term (3 week) diet influenced gene expression at the system level, and that the kibble diet was proinflammatory and the raw red meat diet had anti-inflammatory effects. The comparison of the RMBD and KD in the present study showed that the differentially expressed genes mainly related to immune function, where *CBS* and *PIGR* also have anti-inflammatory effects when upregulated. Our findings support the results of Anderson et al. (104), demonstrating a similar effect over a longer diet intervention. However, more research is needed to verify this observation.

In the present study, genes upregulated in the skin of RMBD fed dogs were found to be related to innate immune function, inflammation and antioxidants, possibly indicating that their innate immunity was enhanced, and that there was less oxidative stress. Thus, RMBDs may have an important role in preventing hypersensitivities and disturbed immunity in puppyhood (105). Since the raw food was served raw, it may have enhanced the passive innate immunity more than the more sterile and processed KD. This has been suggested as a reason for the protective effect of raw meat-based diets in canine *Toxocara canis* infections (106). High-protein diets have been shown to be anti-inflammatory in the skin of mice and they might also prove to be beneficial for dogs suffering from allergic skin conditions (107). Secondary skin infections are common in atopic dogs, and antibiotics are often used when treating them. Antibiotic resistant bacteria are an increasing problem in veterinary medicine (108, 109). If the immunity of the skin could be enhanced through diet at a young age, it might decrease the frequency of antibiotic treatments, although further research is warranted.

Since this was a pilot study, the sample size was limited. To counteract this, two different algorithms were used to analyze the data. However, our results might still in part reflect the individual genetic differences between the dogs. Because these dogs were client-owned, it cannot be ruled out that different environmental factors may have affected the results. The KD and RMBDs had both very different macronutrient profiles and ingredients and their comparison was performed intentionally as a test between two common types of canine diet. Although this complicates the interpretation of the results it nevertheless shows the differential effect that diets had on gene expression. A larger sample size with more controlled diets should be used to validate the results of this study.

## Conclusions

The present study showed that lipid metabolism and differentiation of keratinocytes were possibly altered in the skin of atopic dogs. Additionally, compared to the KD fed group, the gene transcription profile of dogs induced by the RMBD in this study is consistent with an enhancement of innate immunity and decreased oxidative stress and may have an important role in preventing hypersensitivities and a disturbed immunity. As there were two major factors differentiating the diets, processed vs. non-processed and high carbohydrate vs. high fat, further studies must be conducted to determine which, or to what extent these factors influenced the results seen in the present study.

## Data Availability Statement

The RNA-Seq data can be found in the SRA database under accession number SRP110851.

## Ethics Statement

This animal study was reviewed and approved by Animal Experiment Board in Finland (ELLA) (permit number: ESAVI/3244/04.10.07/2013). Written informed consent was obtained from the owners for the participation of their animals in this study.

## Author Contributions

JA, AH-B, and KE contributed conception and design of the study. JA, SZ-L, and KE did laboratory work. JA, SZ-L, KE, AH-B, and HS analyzed and interpreted the data. JA wrote the first draft of the manuscript. SZ-L, KE, AH-B, and HS wrote sections of the manuscript. All authors contributed to manuscript revision, read, and approved the submitted version.

## Conflict of Interest

The authors declare that the research was conducted in the absence of any commercial or financial relationships that could be construed as a potential conflict of interest.

## Supplementary Material

Supplementary Table 1 Composition and analytical constituent of the study kibble diet Hill's Science Plan Canine adult sensitive skin with chicken.

Supplementary Table 2 Composition and analytical constituent of the study raw meat-based food Mush Vaisto diets.

Supplementary Table 3 Differentially expressed transcripts, biological functions, and pathways after the diet intervention in atopic vs. healthy dogs.
