# Supplementary material for: The Effect of Atopic Dermatitis and Diet on the Skin Transcriptome in Staffordshire Bull Terriers
Source: Front Vet Sci. 2020 Oct 16;7:552251. doi: 10.3389/fvets.2020.552251 (PMC7596200; doi:10.3389/fvets.2020.552251)
Supplement: Supplementary Table 1 — Composition and analytical constituent of the study kibble diet Hill's Science Plan Canine adult sensitive skin with chicken. [file Table_1.DOCX]

**Table S1.** Composition and analytical constituent of Hill’s Science Plan Canine adult sensitive skin with chicken^†^ (Hill's Pet Nutrition, Inc., Topeka, Kansas, USA).

| Analytical Constituent | In food | In dry matter |
| --- | --- | --- |
| Protein (%) | 25.3 | 27.5 |
| Fat (%) | 16 | 17.4 |
| Carbohydrate (NFE) (%) | 44.5 | 48.4 |
| Fiber (crude) (%) | 1.3 | 1.4 |
| Ash (%) | 4.9 | 5.3 |
| Moisture (%) | 8 | - |
| Calcium (%) | 0.66 | 0.72 |
| Phosphorus (%) | 0.58 | 0.63 |
| Calcium : Phosphorus | 1.1 | 1.1 |
| Sodium (%) | 0.35 | 0.38 |
| Potassium (%) | 0.64 | 0.7 |
| Omega-3 fatty acids (%) | 1.2 | 1.3 |
| Omega-6 fatty acids (%) | 4.8 | 5.2 |
| Vitamin E (mg) | 60 | 65 |
| Vitamin C (mg) | 7 | 7.6 |
| Beta-carotene (mg) | 0.15 | 0.16 |
| ADDITIVES PER kg: |  |  |
| Vitamin A (IU) | 16000 | 17391 |
| Vitamin D (IU) | 941 | 1023 |
| Iron (mg) | 53.7 | 58.4 |
| Iodine (mg) | 0.9 | 1.0 |
| Copper (mg) | 5.3 | 5.8 |
| Manganese (mg) | 5.6 | 6.1 |
| Zinc (mg) | 111 | 121 |
| Selenium (mg) | 0.15 | 0.16 |

^†^ **Composition (2013):** Rice, maize, poultry meat meal (min. chicken 23%), maize gluten meal, dried whole egg, vegetable oil, flaxseed, digest, animal fat, potassium chloride, salt. The diet is stated as complete diet by the manufacturer.
