# Supplementary material for: The Effect of Atopic Dermatitis and Diet on the Skin Transcriptome in Staffordshire Bull Terriers
Source: Front Vet Sci. 2020 Oct 16;7:552251. doi: 10.3389/fvets.2020.552251 (PMC7596200; doi:10.3389/fvets.2020.552251)
Supplement: Supplementary Table 2 — Composition and analytical constituent of the study raw meat-based food Mush Vaisto diets. [file Table_2.DOCX]

**Table S2.** Composition and analytical constituent of Mush Vaisto diets.

| Analytical Constituent, pork-chicken-lamb^†^ | In food | In dry matter |
| --- | --- | --- |
| Protein (%) | 15.2 | 38 |
| Fat (%) | 20 | 50 |
| Ash (crude) (%) | 4.20 | 10.5 |
| Fiber (crude) (%) | 0.60 | 1.5 |
| Moisture (%) | 60.0 | 0.0 |
| Phosphorus (%) | 0.65 | 1.6 |
| Calcium (%) | 1.09 | 2.7 |
| Calcium : Phosphorus | 1.7 | 1.7 |
| Analyzed ingredients from different batch per kg^§^ | | |
| Omega-3 fatty acids (%) |  | 0.4 |
| Omega-6 fatty acids (%) |  | 3.8 |
| Vitamin A (IU) |  | 143050 |
| Vitamin D (IU) |  | 698 |
| Vitamin E (mg) |  | 46.6 |
| Iron (mg) |  | 123 |
| Iodine (mg) |  | 1.86 |
| Copper (mg) |  | 24.2 |
| Manganese (mg) |  | 8.8 |
| Zinc (mg) |  | 119 |
| Selenium (mg) |  | 0.62 |
| Analytical Constituent, beef-turkey-salmon^‡^ | **In food** | **In dry matter** |
| Protein (%) | 15.0 | 42.5 |
| Fat (%) | 15.8 | 44.8 |
| Ash (crude) (%) | 3.70 | 10.5 |
| Fiber (crude) (%) | 0.80 | 2.3 |
| Moisture (%) | 64.7 | 0.0 |
| Phosphorus (%) | 0.34 | 1.0 |
| Calcium (%) | 0.45 | 1.3 |
| Calcium : Phosphorus | 1.3 | 1.3 |
| Analyzed ingredients from different batch per kg^§^ | | |
| Omega-3 fatty acids (%) |  | 1.1 |
| Omega-6 fatty acids (%) |  | 2.7 |
| Vitamin A (IU) |  | 80890 |
| Vitamin D (IU) |  | 2130 |
| Vitamin E (mg) |  | 54.4 |
| Iron (mg) |  | 82.1 |
| Iodine (mg) |  | 1.64 |
| Copper (mg) |  | 31.5 |
| Manganese (mg) |  | 7.4 |
| Zinc (mg) |  | 79.6 |
| Selenium (mg) |  | 0.73 |

^†^**Composition:** (pork-chicken-lamb): Finnish pork 46% (meat, bone, lung, cartilage, heart, liver), Finnish chicken 29% (meat, bone, gizzard, skin, heart, cartilage, liver), Finnish lamb 20% (bone, meat, lung, cartilage, liver), vegetables 5% (spinach, broccoli, lettuce, cold-pressed sunflower oil), egg < 1%.

^‡^**Composition:** (beef-turkey-salmon): Finnish beef, 47% (rumen, meat, lung, heart, cartilage, liver), Finnish turkey 38% (meat, bone, cartilage), Norwegian salmon 10% (salmon including bones), vegetables 5% (broccoli, lettuce, apple, carrot, cold-pressed sunflower oil, camelina oil). The diets have been stated as complete by the manufacturer.

*Ingredients were analysed by the manufacturer from different food batch and provided to researchers by MUSH Ltd.
